# The role of Tumor Necrosis Factor -Alpha (TNF-α) in bone resorption present in middle ear cholesteatoma

**DOI:** 10.1016/S1808-8694(15)31133-2

**Published:** 2015-10-20

**Authors:** Rodrigo Faller Vitale, Fernando de Andrade Quintanilha Ribeiro

**Affiliations:** 1 Preceptor of Otorhinolaryngology – Otorhinus Clinic and Santa Marcelina Hospital; 2 Adjunct Professor of Otorhinolaryngology – School of Medical Sciences - Santa Casa de São Paulo

**Keywords:** cholesteatoma, tumor necrosis factor, ear middle, bone resorption

## Abstract

**Summary:**

Cholesteatoma may cause bone erosion, with high morbidity and mortality rates. Tumor Necrosis Factor - Alpha (TNF-a) is one of the main cytokines involved in this process. Our goal was to evaluate the role of TNF-a in Bone Resorption and its effect on cholesteatoma.

**Material and Methods:**

analysis and critical literature review.

**Results:**

Different studies have demonstrated that TNF-a is capable of causing bone erosion. It may stimulate the differentiation and maturation of osteoclasts or it may act on the bone matrix, exposing it to the action of the osteoclasts. It is possible to inhibit TNF-a, reducing its effects and prevent bone loss in illnesses such as rheumatoid arthritis, and there has been no specific investigation regarding cholesteatomas. All studies agree on the importance of TNF-a in the bone resorption process present in cholesteatomas, and on the degree of destruction observed; however, there is no consensus as to its location. These differences are probably due to receptor site.

**Conclusion:**

TNF-a, present in cholesteatomas, promotes bone resorption, along with other cytokines (RANKL and IL-1) related to complications.

## INTRODUCTION

Acquired middle ear cholesteatoma was first described by Curveilhier in 1829. It is characterized by the invasion of keratinized stratified squamous epithelium into the tympanic cavity. This epithelium is different from that usually found in the middle ear.^1^

Cholesteatoma may cause bone destruction and result in intratemporal and intracranial complications,^2^ which have high morbidity and mortality rates. This justifies our study of the bone destruction mechanisms in cholesteatoma.

There are numerous theories to explain the destructive properties (erosion) of cholesteatomas. Pressure due to accumulated keratin and other waste was initially proposed as the cause of destruction. A biochemical theory eventually was postulated, in which enzymes and cytokines released by cholesteatomas would cause bone lysis and destruction.^3^

Cytokines are a group of low molecular weight proteins for communication between cells.^4^ Cytokines are released as a result of a variety of stimuli, and interact with their receptors to regulate cell function.^5^ They are closely related to inflammation. The tumor necrosis factor alpha (TNF-α) was discovered by Carswell et al.^6^ in 1975; it is considered one of the main cytokines related to inflammation and immune processes, and operates in various parts of the body. TNF-α is secreted by macrophages, lymphocytes and monocytes mostly as a result of the presence of bacterial lipopolysaccharides (LPS). TNF-α and other cytokines cause bone destruction and remodelling7 in cholesteatomas.

The aim of this paper is to analyze the importance of tumor necrosis factor alpha (TNF-α) in bone resorption and its action in acquired middle ear cholesteatoma based on a review and an analysis of literature.

## REVIEW OF LITERATURE

### Properties of TNF-α

TNF-α is produced by activated macrophages, lymphocytes or monocytes.^8^ The main stimulus for its production are lipopolysaccharides that are part of gram-negative bacterial membranes.^9^ After production and release, TNF-α links to specific receptors called TNF receptors I and II (TNF-R) to produce biological effects. TNF receptors (particularly TNF-RII) may also initiate apoptosis. The mechanism that defines the dominant effect, however, has not been fully clarified.^5^ Thus, the main physiological effect of TNF-α is to promote the immunological and inflammatory response by recruiting neutrophils and monocytes to an infection site followed by their activation. TNF-α therefore has a number of effects in the body.^5^

When released at low concentrations, TNF-α acts on endothelial cells to promote vasodilation and stimulates these cells to secrete a group of leukocyte-chemotaxis cytokines named chemokines. Resulting local inflammation combats infections.^5^ In the hypothalamus TNF-α is an endogenous pyrogen that causes fever. In the liver, TNF-α stimulates the production of acute phase inflammatory proteins and fibrinogen.^5^

TNF-α leads to bone destruction by acting directly on osteoclast differentiation and maturation, and by indirectly exposing the bone matrix.^10^ It does so together with interleukin 1 (IL-1) and RANKL (ligandins for activating NF-κB receptors), which are also abundant in inflammation sites associated with bone destruction. Together, these substances can recruit, differentiate, and activate osteoclasts.^11^ There is, therefore, synergy between TNF-α and RANKL that increases osteoclast function by a cooperation mechanism,^8^,^12^ to which may be added interleukin 1 and 6 (IL-1 and IL-6).^13^,^14^

TNF-α may be inhibited by antagonist solutions containing a soluble portion that block its receptors. Assuma et al.^12^ (1998) observed a roughly 60% reduction of bone loss by using these antagonists. TNF-α may also be inhibited by anti-TNF-α antibodies.^15^

### Action of TNF-α in acquired middle ear cholesteatoma

The first papers that investigated the role of TNF-α in cholesteatoma were published in the beginning of the 1990s.^16^,^17^ Since then many authors have studied the action of TNF-□ on bone resorption in cholesteatoma.^18^

Yan and Huang^11^ (1991) analyzed five experimentally induced cholesteatomas in animals and five surgical fragments. They found that human and experimental cholesteatomas had varying degrees of inflammation, including mononuclear infiltrates (lymphocytes and macrophages) and signs of vascular proliferation. Immunohistochemistry demonstrated TNF-α in granulation tissue. The authors noted that adding TNF-α to bone tissue cultures stimulated the formation of multinucleated cells (osteoclasts) that produced a gap on the bone surface (Howship's lacunae). They concluded that TNF-α is present in granulation tissue adjacent to the cholesteatoma and causes bone resorption^11^.

Marenda and Aufdemorte^19^ (1995) attempted to find TNF-α and other cytokines in surgically collected cholesteatomas, comparing them with normal skin fragments from the external auditory meatus (EAM). TNF-α was found in suprabasal layers of cholesteatomas and in macrophages and fibroblasts; it was not found in the basal layer or in the EAM.

Amar et al.^20^ (1996) studied bone destruction in cholesteatoma by dosing the TNF-α concentration in patients undergoing surgery. They also subdivided cholesteatomas into two groups: local and extended (based on the number of eroded ossicles, the presence of facial canal erosion, exposure of the dura mater and the sigmoid sinus). TNF-αconcentration was significantly higher in more aggressive cholesteatomas. Amar et al.^20^ (1996) concluded that cytokines - TNF-α in particular - are directly involved in bone destruction caused by cholesteatomas, acting as an autocrine growth factor and indirectly stimulating hydrolase lysozymes.

Chung and Yoon^21^ (1998) separated the subepithelial and epithelial portion of cholesteatomas and cultured these tissues. These authors found that epithelial cells only produced interleukins (cytokines similar in function to TNF-α) when associated with the subepithelial tissue. They concluded that substances from the perimatrix possibly stimulate the cholesteatoma matrix to produce IL1, resulting in bone resorption.

Sastry et al.^7^ (1999) dosed serum TNF-α levels in twenty normal patients (control group) and in twenty patients that were to undergo surgery for middle ear cholesteatoma. They collected surgical fragments of cholesteatomas and noted the degree of bone destruction. According to their findings, patients undergoing otological surgery had significantly higher preoperative serum TNF-α levels. They also found differences in serum TNF-α levels depending on the degree of bone destruction.

Akimoto et al.^22^ (2000) studied the role of TNF-α in inflammation in congenital and acquired cholesteatomas. They noted that TNF-α and IL-1 levels are higher in these conditions compared to the skin of the EAM.

Yong-Soo et al.^13^ (2001) also attempted to identify the importance of TNF-α in middle ear inflammation in rats with secretory otitis media induced by injecting Psudomonas aeuroginosas endotoxins. They noted the possibility of inhibiting inflammation. This was done dividing the rats into two groups. Saline solution containing a TNF-α antagonist (sTNF RI) was injected in rats belonging to the first group; rats in the second group received no injection. The authors found that the concomitant injection of sTNF RI inhibited inflammation. According to these authors, a TNF-α antagonist could be an important measure in the treatment of secretory otitis media, avoiding complications and cholesteatomas.

Yetiser et al.^23^ (2002) compared TNF-α and IL-1 levels in sixteen cholesteatoma-free chronic otitis media patients and twenty chronic otitis media patients with cholesteatomas. They found significantly higher levels of IL-1 and TNF-α in the second group, and concluded that bone destruction was mediated by these cytokines.

Alves and Ribeiro^18^ (2004) reviewed the role of cytokines in acquired middle ear cholesteatoma. They found a lack of consensus in literature about the localization of TNF-α in cholesteatoma layers. According to these authors TNF-α acts as a mediator in bone destruction.

## DISCUSSION

Cytokines are proteins formed within cells that, when released, interact with transmembrane cell receptors to produce their biological effect.^4^ Depending on the site of action, they may have an autocrine action (when acting within their mother cell), a paracrine action (when acting on neighboring cells), or an endocrine action (acting at a distance). Each cytokine has multiple actions (pleiotropic effect), operating in synergy or antagonism, such as the synergism between TNF-α and interleukin 1 (IL-1), the main cytokines released in inflammation.^4^,^5^

**Figure 1:**
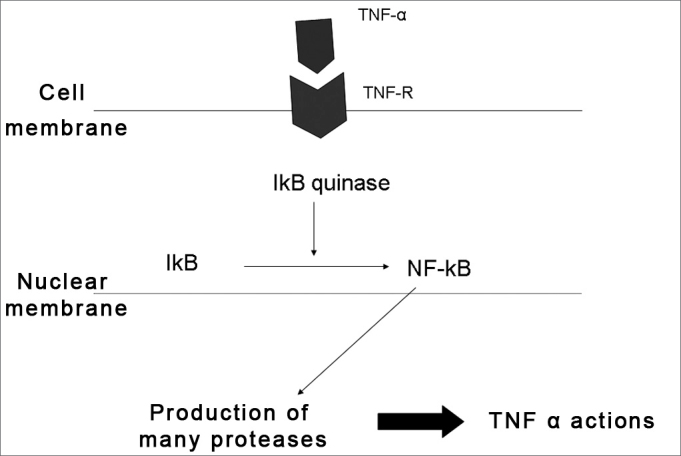
Action of TNF-α on a cell. After linking to its receptor, TNF-α activates NF-κB, which produces and releases a variety of substances involved in inflammation and the immunological response.

TNF-α is produced by activated macrophages or by lymphocytes and monocytes. After linking with its receptors, TNF-α stimulates transcription and production of the IκB kinase enzyme, which in turn produces the nuclear factor κB (NF-κB). When activated, NF-κb acts on the cell nucleus inducing the production of a variety of proteins involved in inflammation and immunological processes responsible for the biological action of TNF-α.^8^ (Figure 1).

Studies on the effect of TNF-α in cholesteatoma started in the beginning of the 1990s, when TNF-α was observed in that condition.16,17 Yan and Huang^11^ (1991) noted that adding a prepared supernatant of cholesteatoma fragments to a bone tissue culture stimulated the formation of multinucleated cells and the appearance of surface lacunae. Multinucleated cells were in fact the osteoclasts found in lacunae (Howship's lacunae) and responsible for bone destruction. It was then demonstrated that cholesteatomas can lead to bone resorption by releasing TNF-α. Blocking TNF-α reduces bone resorption.^8^,^14^,^22^ All of the authors we reviewed agree that TNF-α is one of the main factors responsible for bone resorption and destruction seen in diseases that course together with chronic inflammation.

Osteoclasts are the main cells involved in bone resorption. They originate from monocytes and macrophages and act directly on the bone matrix, causing bone erosion and remodeling. TNF-α, IL-6, IL-1 α or β, interleukin 11 (IL-11), and the macrophage colony stimulating factor (M-CSF) are all responsible for osteoclast differentiation and regulation. These factors, therefore, act directly on bone resorption.^8^,^10^,^12^

Other factors, however, have an indirect action on osteoclasts or on bone-lining cells, by exposing the bone matrix to osteoclasts. These include the parathyroid hormone, prostaglandins, estrogen, IL-11, IL-1 α or β, and TNF- α (which acts both directly and indirectly).^10^,^12^

Osteoblasts release a substance - RANKL - that stimulates differentiation of macrophages into osteoclasts. There is synergy between TNF-α and RANKL in that both cooperate to increase the action of osteoclasts.11 Authors that have studied TNF-α action mechanisms believe that it promotes bone resorption by two pathways (direct and indirect),^8^,^10^,^12^ interacting with other cytokines (RANKL and IL-1) in this process.^11^,^13^

All of the authors we reviewed agree that TNF-α is present in cholesteatomas. There is no consensus, however, about its localization. Alves and Ribeiro^18^ (2004) summarized the differences in opinion. (Table 1) TNF-α is released in all middle ear infections, not only in cholesteatoma.^17^,^23^ In other chronic infections of the ear, however, the bone destruction seen in cholesteatoma is not observed. Chung and Yoon (1998)^21^ noted that interleukin (that stimulates the cholesteatoma matrix to cause bone destruction) is released in the perimatrix. Bone destruction is not seen when the epithelial tissue (matrix) is separated from the subepithelial tissue (perimatrix) in cholesteatoma. The difference may be explained by the presence and distribution of TNF-α receptors in the cholesteatoma epithelium (matrix). Sufficient studies to prove the location of TNF-α receptors in cholesteatoma and the factors that enhance its expression are lacking in literature.

**Table 1:** Location of TNF-α in cholesteatoma, according to various authors.

Authors	Matrix	Perimatrix
Yan and Huang11	Basal layer	
Stratum spinosum	Macrophages	
Marenda and		
Aufdemorte19	Suprabasal	Macrophages
Fibroblasts		
Sastry et al.7	All layers	Negative
Akimoto et al.22	Negative	Positive

Source: Based on work by Alves and Ribeiro18 (2004)

The quantity of TNF-α, and not only the number of receptors, also seems to be related to bone destruction. Various authors have attempted to relate TNF-α levels with intraoperative findings.^7^,^20^,^23^ Cholesteatomas with increased bone destruction had a higher local amount of TNF-α,^7^,^20^,^23^ a finding seen both in acquired and congenital cholesteatomas.^22^ There is, however, only one published paper comparing congenital and acquired cholesteatomas, so this last findings needs to be further substantiated. The plasmatic concentration of TNF-α in cholesteatoma patients with variable degrees of bone destruction is proportionally higher compared to normal patients. A correlation may be established between systemic TNF-α levels and its local behavior.^7^ In future it may become possible to predict the aggressiveness of cholesteatoma through a blood test, predicting the associated degree of bone destruction and the possibility of complications.

Based on these papers, we may conclude that TNF-α is associated with bone destruction and defines its severity.^7^,^20^,^23^ TNF-α is increased both systemically and locally in aggressive cholesteatomas. None of the authors, however, investigated why TNF-α levels are increased. The difference in TNF-α levels may be related to various other factors such as, for instance, duration of the disease, the intensity of local infection, or the presence and distribution of TNF-α receptors in the cholesteatoma matrix. There are, however, insufficient published papers for a more detailed analysis of this hypothesis.

TNF-α is one of the main factors responsible for bone resorption and resulting complications in a variety of diseases. The possibility of interrupting the inflammation seen in such chronic diseases was raised following the discovery of natural inhibitors against TNF-α, which could reduce morbidity and mortality.^24^, ^25^, ^26^ The first large-scale clinical trial of these inhibitors in systemic diseases demonstrated the efficacy of this treatment in reducing bone resorption.27 Studies have focused on inhibiting bone destruction by blocking the action of TNF-α using TNF-α antagonists or anti-TNF-α antibodies.

In periodontitis injection of a solution containing a TNF-α antagonist (s NF RI) inhibits osteoclast formation, reduces inflammatory cell recruitment and reduces bone loss by 60%.^12^,^28^ Thus, TNF-α antagonists can reduce bone destruction.

Anti-TNF-α antibodies can also reduce and postpone bone destruction in rheumatoid arthritis.^27^ Bone resorption may, therefore, be inhibited using two different pathways. There are, however, no such studies applied to cholesteatoma. Based on these findings, there may be in future a medical treatment to inhibit inflammation and control bone destruction, which would reduce the morbidity and mortality of cholesteatoma.

## CONCLUSION

TNF-α is found in cholesteatomas, promoting bone resorption by different routes. It acts on osteoclast differentiation and maturation (direct pathway) and also exposes the bone matrix (indirect pathway). TNF-α, however, is not the only factor involved in bone resorption; it acts in association with interleukin 1 and RANKL. Furthermore, local levels of TNF-α are related with the degree of bone destruction in this disease.
